# Outcomes of left ventricular assist device implantations at Karolinska University Hospital: A retrospective study

**DOI:** 10.1016/j.jhlto.2024.100093

**Published:** 2024-04-08

**Authors:** Abraham Merzo, Magnus Dalén, Ann Hallberg Kristensen, Michael Melin, Jonas Faxén, Robert Edfors, Emil Najjar

**Affiliations:** a Department of Medicine, Karolinska Institutet, Stockholm, Sweden; b Department of Medicine and Cardiology, Malarsjukhuset, Eskilstuna, Sweden; c Center for Clinical Research Sormland, Uppsala University, Eskilstuna, Sweden; d Department of Molecular Medicine and Surgery, Karolinska Institutet, Stockholm, Sweden; e Department of Cardiac Surgery, Karolinska University Hospital, Stockholm, Sweden; f Department of Cardiology, Karolinska University Hospital, Stockholm, Sweden; g Department of Laboratory Medicine, Karolinska Institute, Stockholm, Sweden; h Department of Physiology and Pharmacology, Karolinska Institute, Sweden; i Department of Clinical Sciences, Danderyd University Hospital, Division of Cardiovascular Medicine, Karolinska Institute, Stockholm, Sweden; j Cardiovascular Studies & Pipeline, Medical Affairs & Pharmacovigilance, Pharmaceuticals Bayer AG, Berlin, Germany

**Keywords:** heart failure, HeartMate 3, heart transplantation, left ventricular assist device, mechanical circulatory support

## Abstract

**Background:**

To descriptively present data and outcomes of left ventricular assist device (LVAD) implantations at Karolinska University Hospital.

**Methods:**

Data were collected from consecutive patients (*n* = 44) who were implanted with HeartMate 3 (HM3) at Karolinska University Hospital between 2017 and 2022. The study presents baseline characteristics, clinical course during the inpatient hospital care after implantation, adverse events, and clinical outcomes.

**Results:**

Median intensive care unit stay and hospital stay after HM3 implantation was 8 (interquartile range [IQR] 6; 15) and 28 days (IQR 22; 36), respectively. In total, 73% underwent ramp test at some point after the implantation. Death from all causes within 30 days postimplantation was 5%. A total of 21 patients (48%) underwent right heart catheterization, at a median of 0.5 years (IQR 0.3; 0.7) after implantation, and all exhibited optimally unloaded left ventricles. During the study period, 34% of the patients were transplanted, 5% were explanted, and 16% died with LVAD. In total, 11% and 5% suffered from early and late right ventricle failures, respectively. Acute renal failure affected 46% and 18% had driveline infection. Spontaneous cerebral hemorrhage and cerebral infarction affected 5% and 7% of the study population, respectively. Gastrointestinal bleeding affected 16%. The median LVAD duration was 10 months (IQR 5; 22). The 1-year survival rate with LVAD was 85%, and the 2-year survival rate was 80%.

**Conclusions:**

The results of this low-volume single-center retrospective study on LVAD implantations align with the results of other studies and international registries.

## Background

Heart failure (HF) is a significant and growing health problem affecting over 26 million patients globally, leading to frequent hospitalizations, reduced quality of life, and poor prognosis.^1^, ^2^

In selected patients with advanced left ventricular heart failure (aHF), heart transplantation (Htx) remains the gold standard therapy. However, shortage of heart donors worldwide has led to an increase in left ventricular assist device (LVAD) implantations over the last decade, both as a bridge to transplantation (BTT) and as a destination therapy (DT).^3^, ^4^

Studies have shown that LVAD improves survival, functional capacity, and quality of life. However, the LVAD technology is still constrained by many complications, including right ventricle failure (RVF), infections and hemocompatibility-related adverse events (AEs).^5^, ^6^, ^7^

In Sweden, DT is not approved as a therapy for aHF. The Swedish Evaluation of Left Ventricular Assist Device as Permanent Treatment in End-Stage Heart Failure (ClinicalTrials.gov Identifier: NCT02592499) trial is currently ongoing to compare survival, medium-term benefits, costs, and potential hazards, between guideline-directed LVAD DT and guideline-directed medical therapy.^8^

In the US, over 2,500 LVADs are implanted yearly, while the European Registry for Patients with Mechanical Circulatory Support (EUROMACS) reports at least 500 implantations per year.^9^, ^10^

HeartMate 3 (HM3) (Abbott, Lake Bluff, IL) is currently the only available heart pump globally.^4^ HM3 was tested in the MOMENTUM 3 trial (Multicenter Study of MagLev Technology in Patients Undergoing Mechanical Circulatory Support Therapy with HeartMate 3) and showed that a heart pump can extend survival to 5 years and beyond.^7^

Some evidence suggests that LVAD center volume may impact outcomes.^11^ However, Cowger et al revealed that outcomes of LVAD patients in both very low- and high-volume LVAD centers may be worse compared to low-medium LVAD centers.^12^ The pivotal factors influencing prognosis include multidisciplinary management to facilitate the selection of appropriate LVAD candidates, a systemic approach to surgical and postoperative care, and proper long-term follow-up. These aspects contribute to better outcomes, which could be applied in small LVAD centers as well.^11^, ^13^

Karolinska University in Stockholm, Sweden, is a small LVAD center that has been implanting LVADs since 2006. The purpose of this study is to present data and outcomes of LVAD implantations at Karolinska University Hospital between 2017 and 2022.

## Methods

### Study population

As 1 of the 5 LVAD centers in Sweden, Karolinska University Hospital covers an area of 2.4 million inhabitants. This study included 44 consecutive patients who underwent implantation with HM3, from November 1, 2017 to December 31, 2022. Data from these patients were collected from electronic medical records.

### The definition of adverse events

The definition of AE is in accordance with the Mechanical Circulatory Support Academic Research Consortium.^14^, ^15^ Only the first AE was registered after the implantation. AEs of interest in this study were early RVF (within 30 days after LVAD implantation), late RVF (beyond 30 days after LVAD implantation), acute kidney injury (AKI), driveline infection, cerebral hemorrhage and infarction, gastrointestinal bleeding, and device malfunction. Device malfunction, specifically major device malfunction, occurs when at least 1 component of the LVADs either directly or indirectly causes a state of inadequate circulatory support or death. Only major malfunctions were recorded in accordance with the definitions of AEs.^15^ Minor malfunction is a disorder in the nonpumping and external parts of the LVAD. Most of these disorders are rectifiable without leading to serious outcomes and therefore not included.^16^

To define post-LVAD AKI, the Kidney Disease Improving Global Outcome (KDIGO) criteria were applied due to their comprehensive definition, ensuring a definitive diagnosis. While the definitions of AKI by Mechanical Circulatory Support Academic Research Consortium and KDIGO are similar, the duration of increased serum-creatinine is different. According to KDIGO, the increased serum-creatinine must persist at least 7 days.^15^, ^17^ The estimated glomerular filtration rate (eGFR) was calculated using the equation from the Chronic Kidney Disease Epidemiology Collaboration 2009.^18^

All AEs were presented as the rate of event per patient-year (EPPY).

### Statistical analysis

Using competing outcomes methodology, the cumulative incidence function for each of the 3 mutually exclusive events (death, transplant, and explant) was calculated. The Kaplan-Meier function was used to illustrate patients still alive on LVAD support and censored at transplantation and explantation. The Wilcoxon test was employed to evaluate the statistical significance of hemodynamic parameters and biomarkers before and after LVAD implantation.

SPSS version 27.0.1 (SPSS Inc., Chicago, IL) and R version 4.3.1 (R Foundation for Statistical Computing, Vienna, Austria) were used for statistical analyses. Continuous variables are presented as median and interquartile range (IQR) and categorical variables are reported as numbers (*n*) and proportions (%).

### Ethics

This study complies with the Declaration of Helsinki and was approved by the regional ethical review board (Dnr: 2016/2576-32).

## Results

### Baseline characteristics at implantation

Baseline characteristics at the time of LVAD implantation are presented in Table 1. The median age was 51 years (IQR 44; 60) and 84% were men. The median left ventricular ejection fraction was 15% (IQR 15; 20), and the median of the left ventricle end diastolic diameter was 70 mm (IQR 63; 77). Invasive right heart catheterization was performed within the 3 months before LVAD implantation. The median mean pulmonary arterial pressure was 32 mm Hg (IQR 29; 40), and median pulmonary capillary wedge pressure (PCWP) was 23 mm Hg (IQR 20; 28). Median N-terminal pro brain natriuretic peptide (NT-proBNP) was 4605 ng/liter (IQR 2,825; 8,400). Most of the patients were in INTERMACS (Interagency Registry for Mechanically Assisted Circulatory Support) 3 (66%) before implantation.

**Table 1:** Preimplant Baseline Clinical Characteristics; Median (Interquartile Range) and *n* (%)

*Demographics N* = *44*	
Age (years)	51 (44;60)
Gender (male)	37 (84)
*Medical history*	
Diabetes mellitus	9 (21)
Hypertension	14 (32)
Atrial fibrillation/flutter	21 (48)
Hyperlipidemia	17 (39)
Current smoker	19 (43)
Body mass index (kg/m^2^)	26 (24;28)
Duration of heart failure (years)	3.5 (0.5;9.0)
*Functional status at implantation*	
NYHA III	34 (77)
NYHA IV	10 (23)
INTERMACS 1	3 (7)
INTERMACS 2	10 (23)
INTERMACS 3	29 (66)
INTERMACS 4	2 (5)
*Heart failure etiology*	
Ischemic cardiomyopathy	15 (34)
Dilated cardiomyopathy	21 (48)
Others causes	8 (18)
*LVAD indication*	
BTC	4 (9)
BTD	23 (52)
BTT	12 (27)
DT	5 (11)
*Echocardiographic measurements*	
LVEF (%)	15 (15; 20)
LVEDD (mm)	70 (63; 77)
RVEDD (mm)	47(43; 50)
TAPSE (mm)	14 (11; 15)
*Hemodynamic parameters*	
mPAP (mm Hg)	32 (29; 40)
PCWP (mm Hg)	23 (20; 28)
CVP (mmHg)	10 (5; 15)
CI thermodilution (liter/min/m^2^)	1.6 (1.4; 2.0)
CI Fick (liter/min/m^2^)	1.8 (1.5; 2.2)
*Ergospirometric parameters*	
Peak VO_2_ (ml/kg/min)	13 (11;16)
VE/VCO_2_ slope (liter/liter)	41 (37;50)
*Treatment*	
ACE-I/ARB/ARNI	39 (87)
Beta blocker	42 (96)
MRA	34 (77)
SGLT2i	18 (41)
Warfarin	18 (50)
NOAC	6 (14)
ASA	10 (23)
P2Y12/ADP-inhibitor	4 (9)
ICD	18 (41)
CRT-D	15 (34)
*Laboratory*	
Hemoglobin (g/liter)	137 (122; 141)
NT-proBNP (ng/liter)	4,605 (2,825; 8,400)
eGFR (ml/min/1.73 m^2^)	60 (42; 80)
Bilirubin (µmol/liter)	18 (11; 26)

Abbreviations: ACE-I, angiotensin converter enzyme inhibitor; ARNI, angiotensin receptor-neprilysin inhibitor; ARB, angiotensin receptor blocker; ASA, acetylsalicylic acid; BTC, bridge to candidacy; BTD, bridge to decision; BTT, bridge to transplantation; CI, cardiac index; CRT, cardiac resynchronization therapy; CVP, central venous pressure; DT, destination therapy; eGFR, estimated glomerular filtration rate; HF, heart failure; ICD, implantable cardioverter defibrillator; INTERMACS, Interagency Registry for Mechanically Assisted Circulatory Support; LVAD, left ventricular assist device; LVEDD, left ventricular end diastolic diameter; LVEF, left ventricular ejection fraction; mPAP, mean pulmonary arterial pressure; MRA; mineralocorticoid receptor antagonist; NOAC, nonvitamin K antagonist oral anticoagulantia; NYHA, New York Heart Association; NT-proBNP, N-terminal pro brain natriuretic peptide; P2Y12, receptor for adenosine diphosphate (ADP), i.e., ticagrelor, prasugrel and clopidogrel; PCWP, pulmonary capillary wedge pressure; RVEDD, basal end diastolic right ventricle diameter; SGLT2i, sodium glucose cotransporter 2 inhibitior; SV, stroke volume; TAPSE, tricuspid annular plane systolic excursion; VE/CCO_2_, minute ventilation/carbon dioxide production; VO_2_, oxygen consumption.

In the study population, 48% (*n* = 21) had dilated cardiomyopathy. The median duration of HF before LVAD implantation was 3.5 years (IQR 0.5; 9.0).

The indications for LVAD were DT (11%), BTT (27%), bridge to candidacy (BTC) (9%), and bridge to decision (BTD) (52%). The decision of further therapy strategy is made by Karolinska University Hospital’s LVAD team. Regarding BTD, it includes patients who are critically ill due to aHF, requiring further optimization of specific hemodynamic parameters and who have not yet undergone investigation for Htx. The decision concerning BTT relates to aHF patients that have undergone pretransplantation investigation and have been accepted for Htx by a Heart Transplantation center. Patients with HF who are in a critical condition, and have a short-term mechanical support, are naturally prioritized for transplantation, as “urgent call” cases.

From November 1 to December 31, 2017, only 1 patient underwent LVAD implantation. In 2018, 13 patients received a device. In 2019, 6 patients were implanted with an LVAD, followed by 5 patients in 2020. In 2021, 10 patients received an LVAD, and in 2022, 9 patients received a heart pump. Mechanical aortic valves were replaced with biological aortic valves at the time of implantation.

### In-hospital care and follow-up characteristics

The in-hospital course, HF medication at discharge, echocardiographic findings, and laboratory findings 3 months after discharge are presented in Table 2. The median length of stay at the intensive care unit (ICU) after the LVAD implantation was 8 days (IQR 6; 15). The median hospital stay was 28 days (IQR 22; 36). The median duration of LVAD support for the entire study population was 10 months (IQR 5; 22). In detail, the median duration of LVAD support was 18 months (IQR 5; 31) for the BTC group, 16 months (IQR 5; 24) for the BTD group, 10 months (IQR 6; 11) for the BTT group, and 6 months (IQR 2; 28) for the DT group. The wide IQR in the DT group was due to 1 patient having LVAD for 36 months, supported until the end of the study, while others received LVAD near the end of the study. The median duration of LVAD support for patients who were transplanted was 10 months (IQR 6; 24). The median duration for those who died with LVAD was 3 months (IQR 1; 6), while those who recovered experienced a median duration of 27 months (IQR 23; 29), and for patients who had LVAD until the end of the study, the median duration was 13 months (IQR 5; 24).

**Table 2:** Early Post-LVAD Implantation; Median (Interquartile Range) and *n* (%)

*Inpatient course*	
Days in ICU	8 (6; 15)
Hospital stay, days postimplantation	28 (22; 36)
Death from all causes within 30 days	2 (5)
Duration of LVAD (months)	10 (5; 22)
*Measurements 3 months after discharge*	
NT-proBNP (ng/liter)	1,265 (704; 2,098)
eGFR (ml/min/1.73 m^2^)	76 (55; 99)
Ecocardiographic ramp test within 3 months	25 (57)
Coagucheck	27 (61)
Recommended heart failure treatment at discharge^19^	
ACE-I/ARB/ARNI	31 (82)
Beta blocker	40 (91)
MRA	32 (73)
SGLT2i	15 (34)

Abbreviations: ACE-I, angiotensin converter enzyme inhibitor; ARB, angiotensin receptor blocker; ARNI, angiotensin receptor-neprilysin inhibitor; eGFR, estimated glomerular filtration rate; ICU, intensive care unit; LVAD, left ventricular assist device; MRA; mineralocorticoid receptor antagonist; NT-proBNP, N-terminal pro brain natriuretic peptide; SGLT2i, sodium glucose cotransporter 2 inhibitior.

Death from any cause within 30 days was 5% (*n* = 2). One patient died of septic shock while the other patient died due to severe early RVF, resulting in multiple organ failure.

Three months after discharge, median NT-proBNP decreased significantly to 1,265 ng/liter (IQR 704; 2,098). Among the study population, 73% had done ramp test at some point after implantation.

### Adverse events

The frequencies of AE and the rate of EPPY are presented in Table 3. No discernible trend of increasing or decreasing AEs was observed throughout the study period. Five patients (11%) experienced early RVF, and 2 patients (5%) had late RVF, with rates of 0.096 and 0.038 EPPY, respectively.

**Table 3:** Complications Post-LVAD Implantation *n* (%) and Event per Patient-Year

Early right ventricle failure (≤30 days)	5 (11)	0.096
Late right ventricle failure (>30 days)	2 (5)	0.038
Renal failure (KDIGO) post-LVAD	20 (46)	0.385
Driveline infection	8 (18)	0.157
Cerebral hemorrhage	2 (5)	0.038
Cerebral Infarction	3 (7)	0.058
GI-bleeding post-LVAD implantation	7 (16)	0.135
Device malfunction	1 (2)	0.019

Abbreviations: KDIGO; Kidney Disease Improving Global Outcome; LVAD, left ventricular assist device.

One patient developed severe RVF almost immediately after the implantation, despite careful preoperative assessment of the right ventricle function. Due to RVF, RVAD was initiated through cannulation of the atrium and pulmonary artery with EXCOR cannulas, initially connected to a centrifugal paracorporeal pump and later switched to Berlin Heart EXCOR. Shortly before the shift from centrifugal paracorporeal pump to Berlin Heart EXCOR, the patient received a biological tricuspid valve due to severe tricuspid valve regurgitation. The patient passed away 3 months postimplantation.

Nearly half of the patients developed AKI after implantation. The rate of AKI was 0.39 EPPY. Nevertheless, after 3 months, the renal function either returned to baseline or improved in 26 patients (72%) (*p* < 0,05). In 10 patients (28%), the kidney function declined. Eight patients (18%) suffered from driveline infection at least once during the study period. The rate of EPPY for the first driveline infection was 0.157. Three patients (7%) suffered from cerebral infarction (0.058 EPPY). Two patients (5%) had spontaneous cerebral hemorrhage (0.038 EPPY). Seven patients (16%) had gastrointestinal bleeding with EPPY of 0.135; but none of these AEs were fatal. Only 1 patient experienced a major device malfunction (2%), due to electrostatic discharge, which led to temporary shutdown of the LVAD (0.019 EPPY).

### Outcomes

At the end of the study period, 45% of the patients were still on LVAD, 34% had undergone Htx, 5% had undergone explantation due to cardiac recovery, and 16% died.

In the BTT subgroup, 1 patient (8%) died, 10 patients (83%) underwent Htx, and 1 patient (8%) had LVAD ongoing at the end of the study period. The median waiting time for Htx in the BTT group was 10 months (IQR 7; 11). In the BTD subgroup, 3 patients (13%) were transplanted, 3 patients (13%) died, 2 patients (9%) were explanted, and 15 patients (65%) still had LVAD support. Half of the BTC subgroup was transplanted (*n* = 2), and the other half was still supported with LVAD at the end of the study. In the DT subgroup, 3 patients (60%) died, and 2 patients (40%) were still on LVAD support.

To assess if the ventricles were unloaded optimally (mean right atrial pressure <12 mm Hg and PCWP <18 mm Hg),^20^ right heart catheterization was performed after implantation. The median time from implantation to right heart catheterization was 6.2 (IQR 4.1; 8.6) months. In total, 48% (*n* = 21) underwent right heart catheterization, and in all cases adequate biventricular unloading was seen. Almost all hemodynamic parameters showed significant improvement after LVAD implantation (Table 4).

**Table 4:** Hemodynamic Parameters and Biomarkers; Median (Interquartile Range) and *n* (%)

	Pre-LVAD implantation	Post-LVAD implantation	
*n* (%)	43 (98)	21 (48)	*p*-value

sPAP (mm Hg)	49 (40; 59)	28 (21; 34)	<0.001
mPAP (mm Hg)	32 (29; 40)	16 (12; 21)	<0.001
PCWP (mm Hg)	23 (20; 28)	8 (5; 12)	<0.001
mRAP (mm Hg)	10 (5; 15)	4 (2; 8)	<0.05
CO thermo (liter/min)	3.3 (2.8; 4.1)	4.1 (3.5; 5.2)	<0.05
CO Fick (liter/min)	3.4 (2.9; 4,1)	4.6 (4.2; 5.5)	<0.05
CI thermo (liter/min/m^2^)	1.6 (1.4; 2.0)	2.2 (1.7; 2.5)	0.053
Cl Fick (liter/min/m^2^)	1.8 (1.5; 2.2)	2.5 (1.9; 2.7)	<0.05
SV thermo (ml)	47 (34; 56)	59 (54; 75)	<0.001
SV Fick (ml)	41 (33; 54)	72 (56; 80)	<0.01
NT-proBNP (ng/liter)	4,605 (2,825; 8,400)	1,265 (704; 2,098)	<0.001
eGFR (ml/min/1.73 m^2^)	60 (42; 80)	76 (55; 99)	<0.01

Abbreviations: CI, cardiac index; CO, cardiac output; eGFR, estimated glomerular filtration rate; LVAD, left ventricular assist device; mPAP, mean pulmonary arterial pressure; mRAP, mean right atrial pressure; NT-proBNP, N-terminal pro brain natriuretic peptide; PCWP, pulmonary capillary wedge pressure; SV, stroke volume; sPAP, systolic pulmonary arterial pressure.

**Figure 1:**
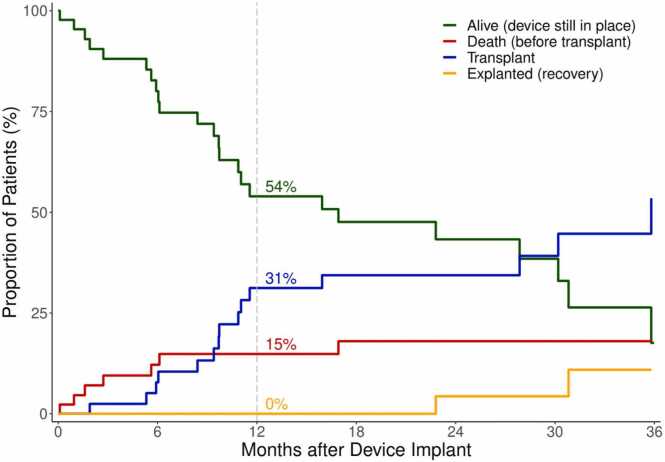
Competitive outcomes in LVAD patients in Karolinska University Hospital. LVAD, left ventricular assist device.

**Figure 2:**
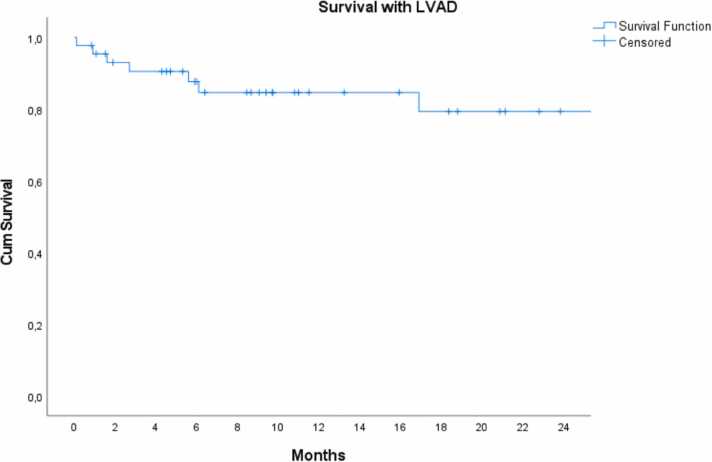
The survival of LVAD patients at Karolinska University Hospital. Transplanted and explanted patients were censored. LVAD, left ventricular assist device.

Figure 1 illustrates the proportion of patients supported with LVAD, and the competing outcomes of death before transplant, transplant, and explant due to recovery. The survival rate with LVAD was 85% at 1 year and 80% at 2 years (Figure 2).

## Discussion

This study reports the clinical characteristics, AEs, and clinical outcomes of the 44 patients implanted with HM3 at Karolinska University Hospital, between 2017 and 2022.

### Baseline characteristics

LVAD patients at Karolinska University Hospital were slightly younger in comparison with other studies. In the INTERMACS annual report from 2020, the mean age was 57 years.^9^ Similarly, EUROMACS presented a median age of 57 years in their third report.^10^ In the MOMENTUM 3, the median age was 62 years in the HM3 cohort.^21^ The age difference might be attributed to the increased percentage of older patients receiving LVADs in the US after the approval of DT. In 2010, the average age increased to 57 years, and the proportion of patients older than 75 years increased approximately to 5% in the US.^22^ Currently, LVAD is not approved as DT in Sweden, and patients are only selected for DT if they are included in the Swedish Evaluation of Left Ventricular Assist Device as Permanent Treatment in End-Stage Heart Failure trial.

Most of our patients were in INTERMACS 3 condition before implantation. According to INTERMACS, in 2019 most of their patients were in INTERMACS 2 prior to implantation.^9^ In EUROMACS, most of the patients were in INTERMACS 1 to 3 before LVAD.^10^ It is vital to select eligible aHF patients who are in an appropriate condition for LVAD implantation.^9^, ^11^, ^23^

Preimplant-reduced renal function is a predictor of higher mortality after LVAD implantation.^24^ This study revealed that the median eGFR significantly increased 3 months after implantation, aligning with the results of previous reports that suggested improvement in eGFR in the following months after LVAD implantation.^25^, ^26^ A subsequent decline may occur over the following several months, although not necessarily returning to pre-LVAD levels.^27^

In this study, NT-proBNP significantly decreased 3 months after implantation. Improved cardiac output and eGFR postimplantation are associated with reduced levels of NT-proBNP. However, it is unknown whether a reduction of NT-proBNP affects outcome after LVAD implantation.^28^

### Inpatient care and follow-up characteristics

The median length of stay at the ICU postimplantation was 8 days (IQR 6; 15) which is in-line with similar reports of EUROMACS.^10^

The median time of LVAD support in our cohort was 10 months. In comparison, INTERMACS reported a median LVAD duration of 54.6 months.^9^ The longer duration of LVAD support in INTERMACS could be explained by the increasing proportion of DT.^4^, ^9^, ^29^

### Adverse events

The greatest advantage with HM3 compared to the older generations of Heart Mates is fewer AE.^21^

Braun et al presented results from the Nordic countries where the rate of RVF in the BTT cohort with HeartMate II (HMII) was 0.2 EPPY.^6^ At Karolinska University Hospital, the incidence of RVF was lower (0.13 EPPY). The difference could be due to different models of Heart Mates.^6^

Even though 46% of the study population developed AKI during the time at the ICU after the LVAD implantation, the early AKI rate per patient-year was lower (0.385) compared to INTERMACS. INTERMACS reported early and late AKI of 0.47 and 0.041 EPPY, respectively.^29^ Using the KDIGO criteria, AKI may be detected at an early stage which may be the reason for the high percentage of AKI.^15^, ^30^ Focusing exclusively on the occurrence of renal replacement therapy in recently implanted patients may lead to a misleading comprehension of the incidence of acute renal failure.^31^, ^32^

This study presented rates of driveline infection (0.157) per patient-year comparable with both the INTERMACS annual report and the Nordic study (0.16 and 0.20, respectively).^6^, ^29^

Reports have confirmed that patients implanted with HM3 have lower incidence of pump thrombosis compared to HMII.^9^, ^10^, ^21^ No patient in this study population had pump thrombosis.

Stroke was less frequent with HM3 in comparison to older generations of LVADs.^21^, ^33^ Karolinska University Hospital had a stroke rate of 0.096 EPPY. INTERMACS reported a stroke rate of 0.29 EPPY within 3 months and 0.069 EPPY after 3 months post-LVAD implantation.^9^, ^29^ The time when the stroke occurred during LVAD support was not taken into consideration in this study.

The rate of gastrointestinal bleeding was 0.135 EPPY. The incidence of this AE in MOMENTUM 3 was 0.25 EPPY.^7^ INTERMACS reported 0.56 and 0.18 EPPY for the rate of early and late GI bleeding.^29^

### Outcomes

In this study, 3 patients died after the implantation at the ICU. One died due to septic shock, 1 due to severe RVF, and 1 due to pneumonia in combination with severe kidney failure. Similarly, previous trials have reported that the main reasons for in-hospital death after LVAD implantation were sepsis, RVF, respiratory failure and multiorgan failure.^28^, ^29^ Early obtained sepsis after LVAD implantation could consequently decrease the survival rate significantly.^30^

In the INTERMACS, the proportion of patients alive on device after 1 year was comparable to our results. The INTERMACS 1-year survival rate in patients implanted with LVAD 2017-2021 was 83% compared to 85% in our study.^29^ The survival rate in INTERMACS has improved in the recent era, and 1 reason could be due to the declined use of HMII after 2015 in favor of HM3.^9^, ^29^ MOMENTUM 3 demonstrated that a heart pump can extend survival to 5 years or beyond.^7^

The survival rate post-LVAD implantation has improved, and the AEs have declined both in Sweden and other countries, mostly due to the advancement in the LVAD technology.^6^, ^29^ The fact that Karolinska University Hospital does not transplant its own patients due to national policies makes LVAD an appealing temporary strategy while waiting for Htx. Due to increasing time on the waiting list, Karolinska University Hospital has seen an increasing number of LVAD implantation as a BTT in recent years.

There is institutional experience and features that may have an impact on outcomes in LVAD centers, such as improved patient selection eligible for LVAD, comprehensive management, including implantation surgery, postoperative care, and ongoing medical follow-up.^11^, ^23^, ^34^, ^35^, ^36^ Small single-center studies emphasize that low-volume centers, such as Karolinska University Hospital, may have the capability to improve the outcomes of LVAD patients.^23^, ^35^, ^36^, ^37^

## Limitations

This study, despite up to 5 years observation period from 1 of Scandinavia’s largest LVAD centers, has notable limitations. Conducted as a single-center retrospective study with a relatively small cohort, caution should be considered when comparing the results to multicenter studies. A larger comparative prospective study between low- and high-volume LVAD centers is required for validation.

## Conclusion

In conclusion, the survival and AE rates of LVAD patients described in this single-center experience at Karolinska University Hospital are in-line with other international reports. The achievement of favorable outcomes with HM3 is facilitated both by the advancement in LVAD technology and a well-established clinical pathway for patients implanted with LVAD. This comprehensive approach includes the inpatient care, surgery, postoperative management, and a well-structured follow-up plan. Thus, low-volume centers, such as Karolinska University Hospital, can maintain optimal care standards for LVAD patients.

## Disclosure statement

E.N.: speaker’s honoraria from Novartis, Astra Zeneca, Bayer, Bristol Myers Squibb. R.E. is an employee of Bayer AG and reports no conflicts related to this work. The other authors have no relationships that could be construed as a conflict of interest.

No funding was received.
